# Short and High-Yielding Synthesis of a Minimalist Diazirine–Alkyne Photo-Cross-Linker and Photoaffinity Labeling Derivatives

**DOI:** 10.1021/acsomega.4c08497

**Published:** 2025-01-22

**Authors:** Dare E. George, Miracle O. Olatunde, Jetze J. Tepe

**Affiliations:** a Department of Chemistry, Michigan State University, East Lansing, Michigan 48823, United States; b Department of Chemistry, University of Virginia, Charlottesville, Virginia 22904, United States

## Abstract

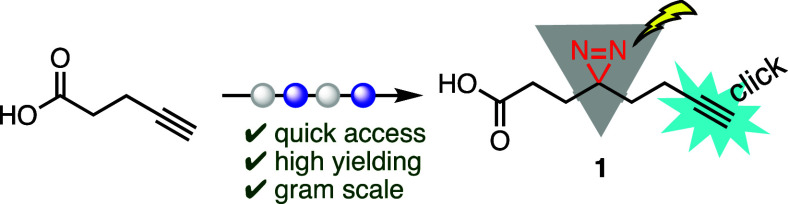

Minimalist photo-cross-linker **1** and its derivatives are extensively utilized in photoaffinity labeling studies. However, obtaining compound **1** in high yield has traditionally required a lengthy synthetic process. In this paper, we present a concise and efficient method to synthesize **1** in just four steps, leveraging the “Normant reagent” as a pivotal component in our strategy. Additionally, we extended our synthetic approach to generate new derivatives of the fully functionalized diazirine tag, providing versatile handles for coupling with other small molecules. This work provides a quick and high-yielding approach to access photo-cross-linker **1** compared to previously reported approaches.

## Introduction

Photoaffinity labeling (PAL) is a powerful technique in biochemical research used to study the interaction of protein and small molecule binding partners. This method involves the use of photoreactive (light-activated) chemical probes that are designed to bind to a target molecule.^1^ Once bound, the probe is activated by exposure to light, which induces the formation of a covalent bond between the probe and its target. This permanent attachment allows for the subsequent identification and analysis of the binding site through methods such as mass spectrometry or western blotting.^2^ Photoaffinity labeling is particularly useful for investigating the molecular details of ligand–receptor interactions, enzyme–substrate relationships, and the identification of unknown proteins within complex biological mixtures.^3^ Its ability to capture transient or weak interactions that might be missed by other techniques makes it a powerful tool in the study of biochemical pathways and drug discovery.

**Figure 1:**
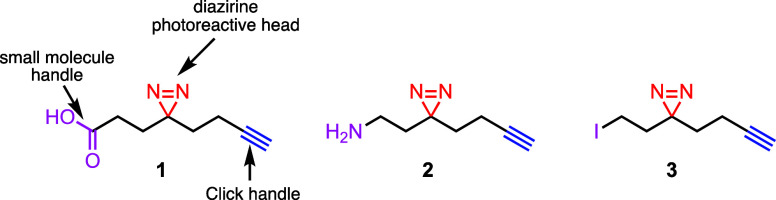
Structure of minimalist alkyne diazirine **1** and derivatives **2** and **3**.

Aliphatic diazirines have attracted enormous attention in the field of PAL over aromatic diazirines, benzophenones, and other photoreactive warheads due to their small size, short irradiation, high specificity, and time needed to generate the reactive carbene. The photoreactive diazirines are designed with a small molecule handle, typically a carboxylic acid, amine, or iodine for coupling with wide arrays of coupling partners. The molecule typically contains the photoreactive diazirine warhead at its center and a terminal alkyne that serves as a click chemistry handle (Figure 1). The minimalist diazirine–alkyne photo-cross-linker **1** and derivatives have been used significantly in the literature for proteomic profiling,^4−10^ photoaffinity-based fragment screening,^11−13^ and ligand ability mapping (Figure 1).^14−17^

**Figure 2:**
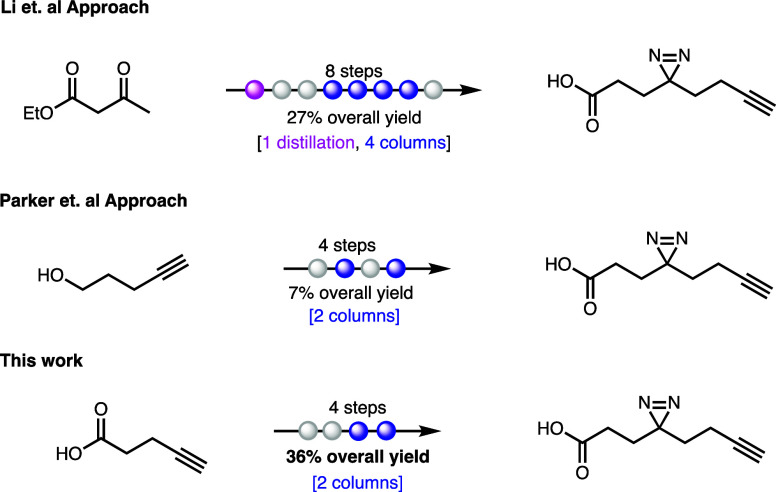
Previous approaches to access minimalist diazirine photo-cross-linker **1** by Li et al.^4^ and Parker et al.^12^ and our approach shown in this work.

Despite the wide use of diazirine photo-cross-linker **1**, it is quite expensive and requires a lot of synthetic effort to access it in good yield (Figure 2). Li et al.^4^ initially reported an eight-step synthesis of **1** in 2013, which requires a Kugelrohr distillation step and at least four silica gel chromatographic columns to access **1** in about 27% overall yield. Even though this approach is mostly used to access compound **1**, it is quite long and requires several purifications. In 2017, Parker et al.^12^ reported a four-step synthesis of **1** in 7% overall yield and only requires two silica gel column chromatographic columns. Although this approach is expedited compared to the Li et al. approach, it suffers from a drastic reduction in overall yield with almost 3-fold loss in the overall yield. These limitations highlight the need for a high-yielding, expedited approach to synthesize photo-cross-linker **1**.

## Results and Discussion

**Scheme 1:**
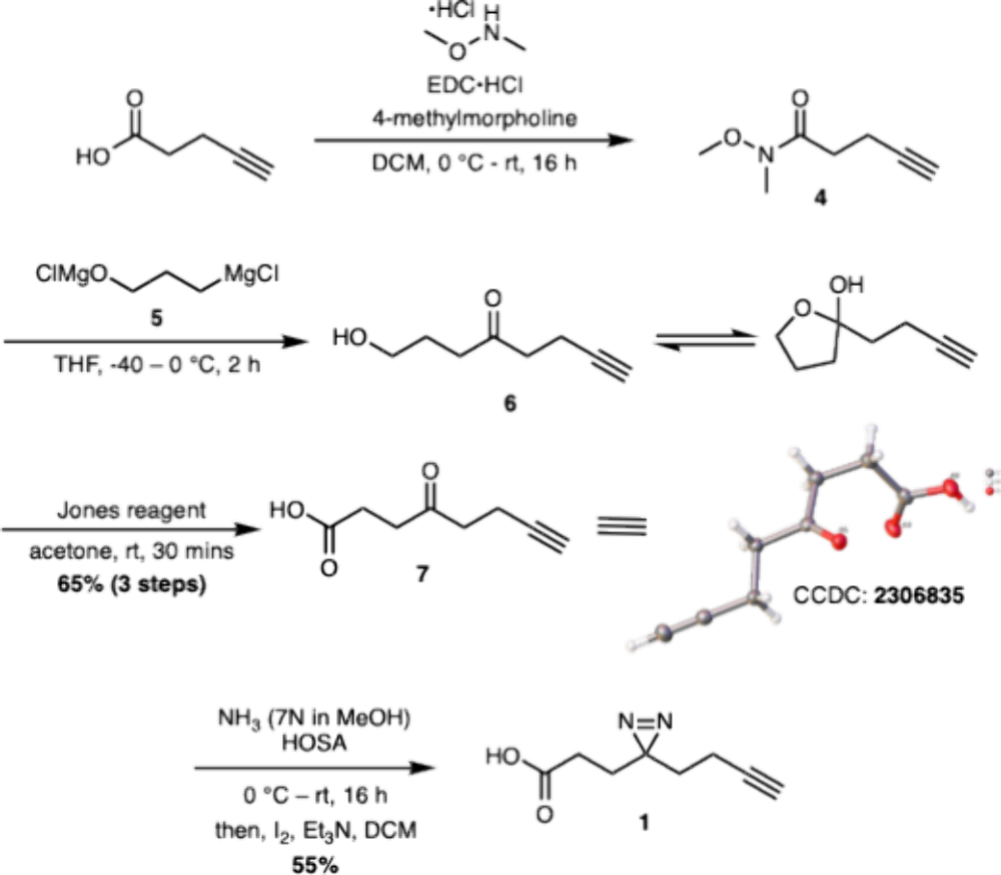
Synthesis of Diazirine Photo-Cross-Linker **1**

We report a new approach to access diazirine photo-cross-linker **1** and new derivatives of the molecule. This new approach allowed the synthesis of **1** in the gram scale and only four steps with an overall yield of 36% using only two silica gel chromatographic column purifications (Scheme 1). We started our synthesis from commercially available 4-pentynoic acid, which was converted to Weinreb amide **4**. The key approach in this synthesis involves the conversion of amide **4** to keto-alcohol **6** using the Normant reagent. The Normant reagent **5** can be prepared in situ from 3-chloropropan-1-ol as originally reported by Normant et al. in 1978.^18^ The keto-alcohol exists in both the linear form and the cyclic hemiacetal. Jones reagent oxidation of the mixture gave the carboxylic acid **7** in 65% yield over three steps and at this step, and the first silica gel column chromatography was performed to purify carboxylic acid **7** and its ester dimer **7b** (see the Experimental Section and the Supporting Information). Compound **7** was then subjected to standard diazirination conditions using an ammonia solution in methanol and hydroxylamine-*O*-sulfonic acid (HOSA) to yield the diaziridine intermediate. This intermediate was subsequently oxidized with iodine and triethylamine to afford diazirine photo-cross-linker **1** in 55% yield.

**Scheme 2:**
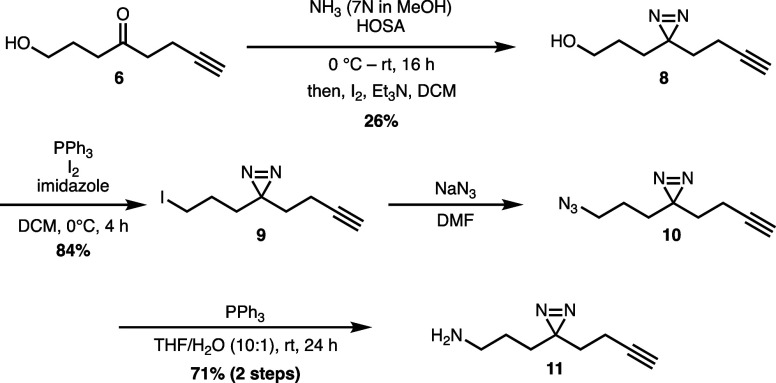
Synthesis of Alkyl Iodide **9** and Amine **11** Derivatives

Following the synthesis of diazirine **1**, we envisioned using intermediate **6** to access other derivatives of probe **1**, particularly the alkyl iodide **9** and amine **11** derivates. This would offer versatility in using this approach to access not only the diazirine probe with a carboxylic acid handle but also with other functional groups for coupling with various small molecules. Starting with compound **6**, we converted the ketone to a diazirine using the same conditions as for the synthesis of compound **1**, yielding compound **8** in only 26% (Scheme 2). The conversion of alcohol **8** to iodide **9** was carried out with iodine in the presence of triphenylphosphine and imidazole to give the desired product in 84%. Compound **9** could be used in a fashion similar to **3** for alkylation of small molecules containing an O, N, or S nucleophile. The amine derivative **11** was synthesized by first treating iodide **9** with sodium azide in DMF to produce compound **10**. Staudinger reduction of **10** with triphenylphosphine at room temperature provided desired amine **11** in 71% yield over two steps.

## Conclusions

In conclusion, we have developed a concise and high-yielding method for synthesizing diazirine photo-cross-linker **1**. This approach was also extended to synthesize alkyl iodide **9** and amine **11** derivatives, offering a versatile means for functionalizing various small molecules with diazirine tags.

## Experimental Section

### General Information

All flasks were oven-dried overnight and cooled under nitrogen. Reactions were carried out under a nitrogen atmosphere unless stated otherwise. All chemicals and solvents were purchased from commercial sources and used without further purification unless otherwise mentioned. Infrared spectra were recorded on a Jasco Series 6600 FTIR spectrometer. The mass spectrometer ionization method was ESI with a quadrupole detector. ^1^H and ^13^C{^1^H} NMR spectra were recorded on a 400 MHz spectrometer. Chemical shifts were reported relative to the residue peaks of the solvent: CDCl_3_: 7.26 ppm for ^1^H and 77.0 ppm for ^13^C. Signal multiplicities were given as s (singlet), br (broad), d (doublet), t (triplet), dd (doublet of doublet), and m (multiplet). Reaction carried out at room temperature (rt) implies a temperature range usually between 20 and 22 °C.

#### *N*-Methoxy-*N*-methylpent-4-ynamide (**4**)

To a solution of the carboxylic acid (7.94 g, 80 mmol) in CH_2_Cl_2_ (200 mL) was added MeNHOMe·HCl (8.76 g, 88 mmol) and 4-methylmorpholine (9.77 mL, 88 mmol) at 0 °C. EDC·HCl (16.87 g, 88 mmol) was then added at the same temperature, and the resulting reaction mixture was stirred at room temperature for 16 h. The reaction mixture was quenched with 1 M HCl solution (88 mL), and the organic layer was extracted with DCM (3 × 100 mL). The combined organic layers were washed with water (100 mL) followed by brine (100 mL) and dried over sodium sulfate. The organic solvent was removed under reduced pressure to give the Weinreb amide **4** as a light-yellow liquid (11.29 g, quantitative). ^1^H NMR (400 MHz, CD_2_Cl_2_) δ 3.67 (s, 3H), 3.14 (s, 3H), 2.65 (t, *J* = 7.4 Hz, 2H), 2.54–2.39 (m, 2H), 2.01 (t, *J* = 2.7 Hz, 1H). ^13^C NMR (101 MHz, CD_2_Cl_2_) δ: 172.5, 83.9, 68.7, 61.6, 32.3, 31.4, 14.0. FTIR (neat, cm^–1^): 3291, 2939, 2120, 1657, 1422, 1387. HRMS (ESI-TOF) *m*/*z*: [M + H]^+^ calcd for C_7_H_12_NO_2_^+^ 142.0863; found 142.0869.

#### 1-Hydroxyoct-7-yn-4-one (**6**)

3-Chloro-1-propanol (6.6 mL, 78.6 mmol) was dissolved in anhydrous THF (10 mL) and cooled to −15 °C. Then, isopropylmagnesium chloride (2.0 M in THF, 39.3 mL, 78.6 mmol) was added dropwise. The solution was allowed to stir for 20 min at −15 °C, and the solution was cannulated slowly over 1 h to a solution of Mg(s) (3.06 g, 125.8 mmol) and 1,2-dibromoethane (0.30 mL) in anhydrous THF (25 mL) at reflux. After complete cannulation, additional 1,2-dibromoethane (0.30 mL) was added, and the solution was further stirred at reflux for 2 h. The solution was then cooled to room temperature and cannulated into a solution of Weinreb amide (4.44 g, 31.45 mmol) at −40 °C over 1 h. The reaction was warmed to 0 °C and stirred for an additional 1 h. The reaction was quenched with 1 M NH_4_Cl (100 mL) and extracted with ethyl acetate (3 × 100 mL). The combined organic layer was washed with brine (200 mL), dried over sodium sulfate, decanted, and then concentrated to give the crude product. The crude product was used directly without any further purification. ^1^H NMR (400 MHz, CDCl_3_) δ 3.64 (q, *J* = 5.9 Hz, 2H), 2.70 (t, *J* = 7.0 Hz, 2H), 2.58 (t, *J* = 7.2 Hz, 2H), 2.50–2.41 (m, 2H), 1.96 (t, *J* = 2.7 Hz, 1H), 1.89–1.82 (m, 2H). ^13^C{^1^H} NMR (101 MHz, CDCl_3_) δ: 209.0, 83.0, 68.8, 62.0, 41.3, 39.4, 26.4, 13.0. FTIR (neat, cm^–1^): 3499, 3289, 2967, 2110, 1709. HRMS (ESI-TOF) *m*/*z*: [M + H]^+^ calcd for C_8_H_13_O_2_^+^ 141.0910; mass not found.

#### 4-Oxooct-7-ynoic acid (**7**)

Alcohol **6** (0.99 g, 7.08 mmol) was dissolved in acetone (10 mL), and the mixture was cooled to 0 °C. A freshly prepared solution of the Jones reagent (5.7 mL, 14.2 mmol) was added slowly. The reaction was stirred for 30 min and then quenched with isopropyl alcohol until the solution changed from orange to a deep green color. The reaction was diluted with water (50 mL) and extracted with ethyl acetate (3 × 50 mL). The organic layers were combined, washed with water (50 mL), dried over sodium sulfate, decanted, and then concentrated to give the crude product. The product was purified with CombiFlash chromatography (silica gel, 20–40 μm, gradient 0–50% ethyl acetate/hexane) to give the product as a white solid (0.71 g, 65%). ^1^H NMR (400 MHz, CDCl_3_) δ 2.80–2.64 (m, 6H), 2.51–2.47 (m, 2H), 1.97 (t, *J* = 2.7 Hz, 1H). ^13^C{^1^H} NMR (101 MHz, CDCl_3_) δ: 206.3, 178.5, 82.8, 68.8, 41.3, 36.8, 27.7, 12.9. FTIR (neat, cm^–1^): 3290, 2923, 2110, 1702. HRMS (ESI-TOF) *m*/*z*: [M – H]^−^ calcd for C_8_H_9_O_3_^–^ 153.0557; found 153.0583.

#### 4-Oxooct-7-yn-1-yl-4-oxooct-7-ynoate (**7b**)

Compound **7b** was isolated as a side product from Jones oxidation of alcohol **6** to carboxylic acid **7**. Compound **7b** (0.075 g, 19%). ^1^H NMR (400 MHz, CDCl_3_) δ 4.07 (t, *J* = 6.3 Hz, 2H), 2.78–2.61 (m, 6H), 2.57 (t, *J* = 6.5 Hz, 2H), 2.51 (t, *J* = 7.2 Hz, 2H), 2.48–2.39 (m, 4H), 1.95 (t, *J* = 2.3 Hz, 2H), 1.90 (q, *J* = 6.7 Hz, 2H). ^13^C NMR (101 MHz, CDCl_3_) δ: 207.4, 206.5, 172.6, 83.0, 82.9, 68.8, 68.8, 63.7, 41.3, 41.3, 38.8, 37.0, 27.8, 22.6, 12.9, 12.9. FTIR (neat, cm^–1^): 3281, 2923, 2120, 1714, 1631. HRMS (ESI-TOF) *m*/*z*: [M + H]^+^ calcd for C_20_H_20_O_4_Na^+^ 299.1254; found 299.1261.

#### 3-(3-(But-3-yn-1-yl)-3*H*-diazirin-3-yl)propanoic Acid (**1**)

A 100 mL round-bottom flask was charged with ketone-carboxylic acid **6** (1.50 g, 9.73 mmol) and cooled to 0 °C. Then, NH_3_ (7.0 N in methanol, 25 mL) was added, and the reaction was sealed and stirred at 0 °C for 1 h. Then, hydroxylamine-*O*-sulfonic acid, HOSA (1.32 g, 11.7 mmol), was added in three portions over 3 h, 0.44 g of HOSA per portion every 1 h while maintaining the reaction temperature at 0 °C. After complete addition, the reaction was allowed to stir overnight. The solvent was then evaporated under reduced pressure to remove the excess ammonia, and the white solid was suspended in methanol (20 mL). The solid was filtered to remove the inorganics and washed with methanol. The filtrate was concentrated under reduced pressure and then redissolved in methanol (10 mL) and triethylamine (5 mL). The solution was cooled to 0 °C and covered with aluminum foil to keep the reaction dark. Iodine was added in portions until a reddish-brown color persisted for about 30 min, indicating the full oxidation of diaziridine to diazirine. The solution was diluted with ethyl acetate (100 mL) and then washed with 1 M HCl (50 mL), Na_2_S_2_O_3_ (2 × 50 mL), and brine (50 mL). The ethyl acetate layer was collected, dried over sodium sulfate, decanted, and concentrated. The reaction was purified with CombiFlash chromatography (silica gel, 20–40 μm, gradient 0–20% ethyl acetate/hexane) to give the product as a clear semisolid compound (0.88 g, 55%). ^1^H NMR (400 MHz, CDCl_3_) δ 2.18 (t, *J* = 7.7 Hz, 2H), 2.07–1.96 (m, 3H), 1.81 (t, *J* = 7.7 Hz, 2H), and 1.66 (t, *J* = 7.2 Hz, 2H). ^13^C{^1^H} NMR (101 MHz, CDCl_3_) δ: 178.3, 82.5, 69.3, 32.2, 28.1, 27.7, 27.4, 13.2. FTIR (neat, cm^–1^): 3293, 2933, 2105, 1702, 1587. HRMS (ESI-TOF) *m*/*z*: [M – H]^−^ calcd for C_8_H_9_N_2_O_2_^–^ 165.0670; found 165.0717.

#### 3-(3-(But-3-yn-1-yl)-3*H*-diazirin-3-yl)propan-1-ol (**8**)

1-Hydroxyoct-7-yn-4-one (2.15 g) was dissolved in ammonia solution (7 N in methanol, 25 mL) in a sealed tube and was allowed to stir at 0 °C to 1 h. Then, hydroxylamine-*O*-sulfonic acid, HOSA (2.08 g, 18.4 mmol), was added in three portions at 1 h intervals, 0.693 g of HOSA per portion every 1 h while maintaining the reaction temperature at 0 °C. After complete addition, the reaction was allowed to stir overnight. The resulting slurry was evaporated to dryness and resuspended in methanol (30 mL). The solid particle was filtered and washed several times with methanol; the resulting filtrate was concentrated and redissolved in methanol (10 mL) and Et_3_N (4 mL). The solution was cooled to 0 °C and covered with aluminum foil to keep the reaction dark. Iodine was added portion-wise until a dark brown color persisted. The solution was diluted with ethyl acetate (100 mL) and then washed with Na_2_S_2_O_3_ (2 × 50 mL) and brine (50 mL). The ethyl acetate layer was collected, dried over sodium sulfate, decanted, and concentrated. The reaction was purified with CombiFlash chromatography (silica gel, 20–40 μm, gradient 0–20% ethyl acetate/hexane) to give the product as a yellow oil (0.61 g, 26%). ^1^H NMR (400 MHz, CDCl_3_) δ 3.55 (t, *J* = 6.4 Hz, 2H), 2.11 (s, 1H), 2.04–1.92 (m, 3H), 1.69–1.58 (m, 2H), 1.56–1.46 (m, 2H), 1.40–1.27 (m, 2H). ^13^C{^1^H} NMR (101 MHz, CDCl_3_) δ: 82.8, 69.2, 61.7, 32.3, 28.9, 28.0, 26.7, 13.3. FTIR (neat, cm^–1^): 3390, 3296, 2941, 2109, 1584. HRMS (ESI-TOF) *m*/*z*: [M + H]^+^ calcd for C_8_H_13_N_2_O^+^ 153.1022; found 153.1021.

#### 3-(But-3-yn-1-yl)-3-(3-iodopropyl)-3*H*-diazirine (**9**)

Iodine (1.47 g, 5.79 mmol) was added to a solution of imidazole (0.79 g, 11.6 mmol) and triphenylphosphine (1.52 g, 5.79 mmol) in CH_2_Cl_2_ (12 mL) at 0 °C. The solution was stirred for 5 min, and alcohol **8** (0.59 g, 3.86 mmol) in CH_2_Cl_2_ (3 mL) was added slowly. The reaction mixture was stirred for 4 h with the exclusion of light. The reaction was then quenched by the addition of an aqueous Na_2_S_2_O_3_ solution (20 mL). The aqueous layer was extracted with DCM (2 × 20 mL). The combined organic layers were washed with brine (20 mL) and dried with anhydrous Na_2_SO_4_. The solvents were removed in vacuo, and the crude product was purified with CombiFlash chromatography (silica gel, 20–40 μm, gradient 0–2% ethyl acetate/hexane) to give the alkyl iodide as a clear oil (0.85 g, 84%). ^1^H NMR (400 MHz, CDCl_3_) δ 3.14 (t, *J* = 6.6 Hz, 2H), 2.12–1.97 (m, 3H), 1.74–1.53 (m, 6H). ^13^C{^1^H} NMR (101 MHz, CDCl_3_) δ: 82.6, 69.3, 33.5, 32.3, 27.7, 27.4, 13.3, 5.2. FTIR (neat, cm^–1^): 3295, 2925, 2120, 1584. HRMS (ESI-TOF) *m*/*z*: [M + H]^+^ calcd for C_8_H_12_IN_2_^+^ 263.0040; mass not found.

#### 3-(3-Azidopropyl)-3-(but-3-yn-1-yl)-3*H*-diazirine (**10**)

Alkyl iodide **9** (0.30 g, 1.14 mmol) was dissolved in DMF (2 mL), and sodium azide (0.11 g, 1.71 mmol) was added. The reaction was heated to 70 °C and stirred in the dark for 5 h. After complete conversion, the reaction mixture was concentrated and used for the next step without further purification.

#### 3-(3-(But-3-yn-1-yl)-3*H*-diazirin-3-yl)propan-1-amine (**11**)

Crude diazirine azide **10** (1.14 mmol) was dissolved in THF (10 mL) and H_2_O (2 mL) at room temperature. Triphenylphosphine (0.48 g, 1.94 mmol) was added, and the reaction mixture was stirred at room temperature overnight. The reaction was diluted with ethyl acetate (20 mL) and was extracted with 1 M HCl (2 × 10 mL). The aqueous layer was neutralized with 2 M NaOH (10 mL), and the product was extracted with ethyl acetate (20 mL). The organic layer was dried over sodium sulfate, decanted, and concentrated to give the amine as a clear oil (0.12 g, 71%). ^1^H NMR (400 MHz, CDCl_3_) δ 2.66 (t, *J* = 7.0 Hz, 2H), 2.09–1.91 (m, 3H), 1.64 (t, *J* = 7.3 Hz, 2H), 1.51–1.47 (m, 2H), 1.42 (s, 2H), 1.33–1.20 (m, 2H). ^13^C{^1^H} NMR (101 MHz, CDCl_3_) δ: 82.8, 69.1, 41.5, 32.3, 30.0, 28.0, 27.8, 13.3. FTIR (neat, cm^–1^): 3295, 2929, 2120, 1582. HRMS (ESI-TOF) *m*/*z*: [M + H]^+^ calcd for C_8_H_14_N_3_^+^ 152.1182; found 152.1184.

## Data Availability

The data underlying this study is available in the published article and its Supporting Information.

## Supporting Information Available

Experimental procedures, characterization data, X-ray crystal data and experiments, and NMR spectra (^1^H and ^13^C NMR) (PDF)

## Accession Codes

CDCC 2306835 contains the supplementary crystallographic data for this paper. These data can be obtained free of charge via www.ccdc.cam.ac.uk/data_request/cif, or by emailing data_request@ccdc.cam.ac.uk, or bycontacting The Cambridge Crystallographic Data Centre, 12 Union Road, Cambridge CB2 1EZ, UK; fax: + 44 1223 336033
