# Supplementary material for: Short and High-Yielding Synthesis of a Minimalist Diazirine–Alkyne Photo-Cross-Linker and Photoaffinity Labeling Derivatives
Source: ACS Omega. 2025 Jan 22;10(4):3622–6. doi: 10.1021/acsomega.4c08497 (PMC11800027; doi:10.1021/acsomega.4c08497)
Supplement: Supplementary file 1 — ao4c08497_si_001.pdf [file ao4c08497_si_001.pdf]

A short and high yielding synthesis of minimalist diazirine-alkyne photo-crosslinker and photo-affinity labelling (PAL) derivatives

Dare E. George[a,b], Miracle O. Olatunde[b], and Jetze J. Tepe[b]*

[a]*Department of Chemistry, Michigan State University, East Lansing, Michigan 48823, United States.*
[b]*Department of Chemistry, University of Virginia, Charlottesville, Virginia, 22904, United States.*

Supporting Information

Table of Contents

Crystal Data and Experimental 3

^{1}H and ^{13}C{^{1}H} NMR Spectra of synthesized compounds. 11

Crystal Data and Experimental

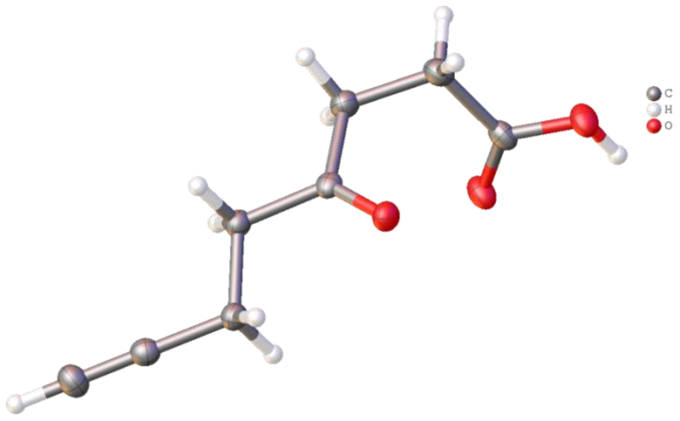

Figure S1: X-ray crystal structure of compound 7

Experimental. Single colourless needle-shaped crystals of **JJT1123B** used as received. A suitable crystal with dimensions 0.28 × 0.18 × 0.13 mm^3 was selected and mounted on a nylon loop with paratone oil on a XtaLAB Synergy, Dualflex, HyPix diffractometer. The crystal was kept at a steady T = 100.00(10) K during data collection. The structure was solved with the ShelXT (Sheldrick, 2015) solution program using dual methods and by using Olex2 1.5 (Dolomanov et al., 2009) as the graphical interface. The model was refined with ShelXL 2018/3 (Sheldrick, 2015) using full matrix least squares minimisation on F^2.

Crystal Data. $C_8H_{10}O_3$, M_r = 154.16, triclinic, P-1 (No. 2), a = 5.68118(11) Å, b = 7.3904(2) Å, c = 9.9783(3) Å, a = 97.838(2)°, b = 106.430(2)°, g = 91.0103(19)°, V = 397.391(17) Å^3, T = 100.00(10) K, Z = 2, Z' = 1, m(Cu K_a) = 0.824, 4833 reflections measured, 1605 unique (R_{int} = 0.0196) which were used in all calculations. The final wR_2 was 0.0788 (all data) and R_1 was 0.0317 (I≥2 s(I)).

**Compound**	**JJT1123B**
Formula	$C_8H_{10}O_3$
CCDC	2306835
$D_{calc.}$/ g cm^{-3}	1.288
m/mm^{-1}	0.824
Formula Weight	154.16
Colour	colourless
Shape	needle-shaped
Size/mm^3	0.28×0.18×0.13
T/K	100.00(10)
Crystal System	triclinic
Space Group	P-1
a/Å	5.68118(11)
b/Å	7.3904(2)
c/Å	9.9783(3)
a/°	97.838(2)
b/°	106.430(2)
g/°	91.0103(19)
V/Å^3	397.391(17)
Z	2
Z'	1
Wavelength/Å	1.54184
Radiation type	Cu K_a
Q_{min}/°	4.671
Q_{max}/°	80.055
Measured Refl's.	4833
Indep't Refl's	1605
Refl's I≥2 s(I)	1528
R_{int}	0.0196
Parameters	102
Restraints	0
Largest Peak	0.223
Deepest Hole	-0.170
GooF	1.050
wR_2 (all data)	0.0788
wR_2	0.0777
R_1 (all data)	0.0330
R_1	0.0317

Structure Quality Indicators

**Reflections:**	d min (CuKα) 2Θ=160.0° 0.78	I/σ(I) 36.3	Rint m=3.01 1.96%	Full 135.4° 93% to 160.0° 97.1
**Refinement:**	Shift 0.000	Max Peak 0.2	Min Peak -0.2	GooF 1.050

A colourless needle-shaped crystal with dimensions 0.28×0.18×0.13 mm^3 was mounted on a nylon loop with paratone oil. Data were collected using a XtaLAB Synergy, Dualflex, HyPix diffractometer equipped with an Oxford Cryosystems low-temperature device, operating at T = 100.00(10) K.

MSU Data were measured using *w* scans using Cu K_α radiation (micro-focus sealed X-ray tube, ? kV, ? mA). The total number of runs and images was based on the strategy calculation from the program CrysAlisPro system (CCD 43.94a 64-bit (release 20-10-2023)). The achieved resolution was Q = 80.055.

Cell parameters were retrieved using the CrysAlisPro 1.171.43.92a (Rigaku OD, 2023) software and refined using CrysAlisPro 1.171.43.92a (Rigaku OD, 2023) on 3409 reflections, 71 % of the observed reflections. Data reduction was performed using the CrysAlisPro 1.171.43.92a (Rigaku OD, 2023) software which corrects for Lorentz polarization. The final completeness is 97.10 out to 80.055 in Q CrysAlisPro 1.171.43.92a (Rigaku Oxford Diffraction, 2023) Numerical absorption correction based on gaussian integration over a multifaceted crystal model Empirical absorption correction using spherical harmonics, implemented in SCALE3 ABSPACK scaling algorithm.

The structure was solved in the space group *P*-1 (# 2) by using dual methods using the ShelXT (Sheldrick, 2015) structure solution program.

CCDC 2306835 contains the supplementary crystallographic data for this paper. The data can be obtained free of charge from The Cambridge Crystallographic Data Centre via www.ccdc.cam.ac.uk/structures.

There is a single formula unit in the asymmetric unit, which is represented by the reported sum formula. In other words: Z is 2 and Z' is 1. The moiety formula is C8 H10 O3.

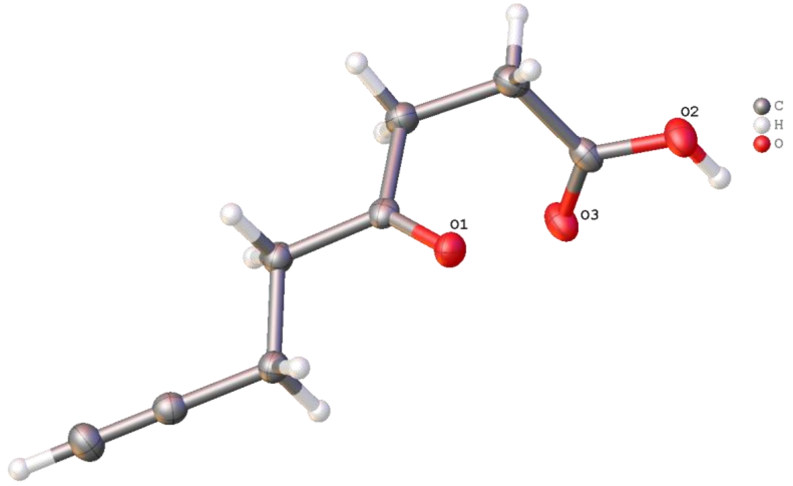

Figure S2: Drawing of compound at 50% ellipsoids showing labeling of hetero-atoms atoms.

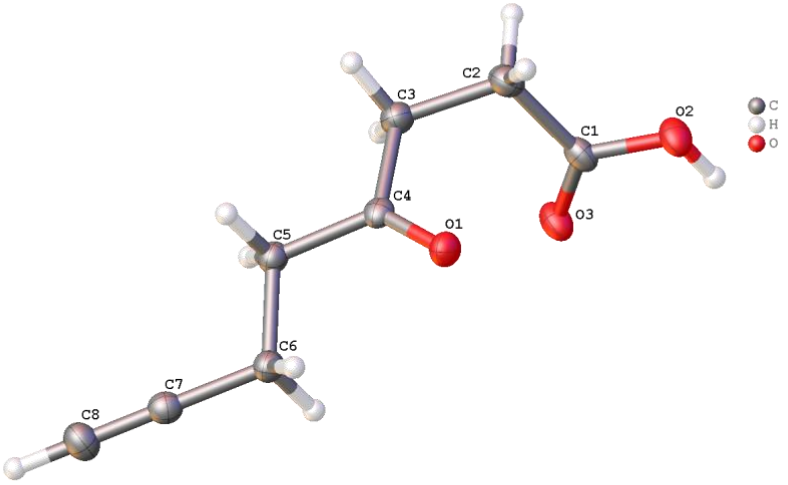

Figure S3: Drawing of compound at 50% ellipsoids showing labeling of all non-hydrogen atoms.

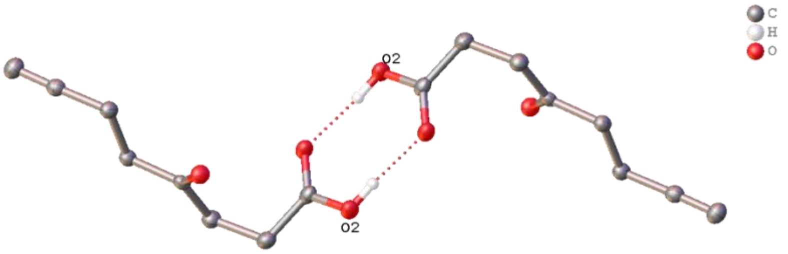

Figure S4: The following hydrogen bonding interactions with a maximum D-D distance of 2.9 Å and a minimum angle of 120 ˚ are present in **JJT1123B**: O2–O3_*1: 2.649 Å.*

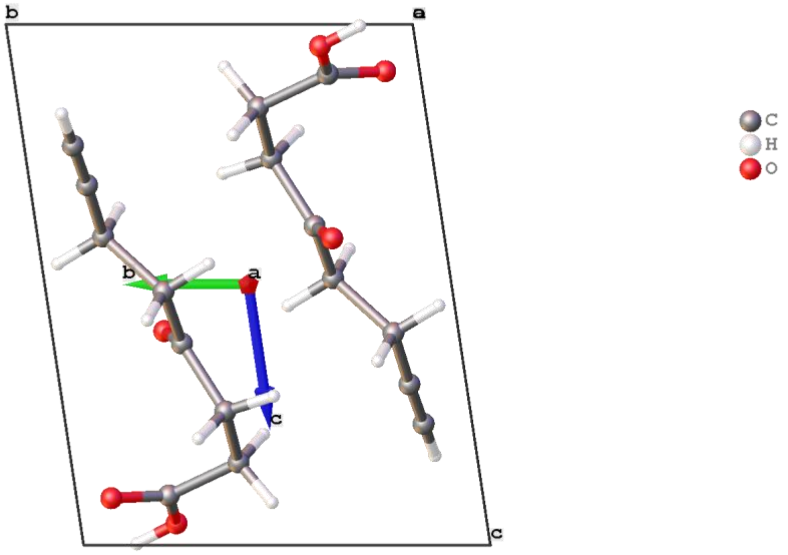

Figure S5: Packing diagram of JJT1123B viewed along the a axis

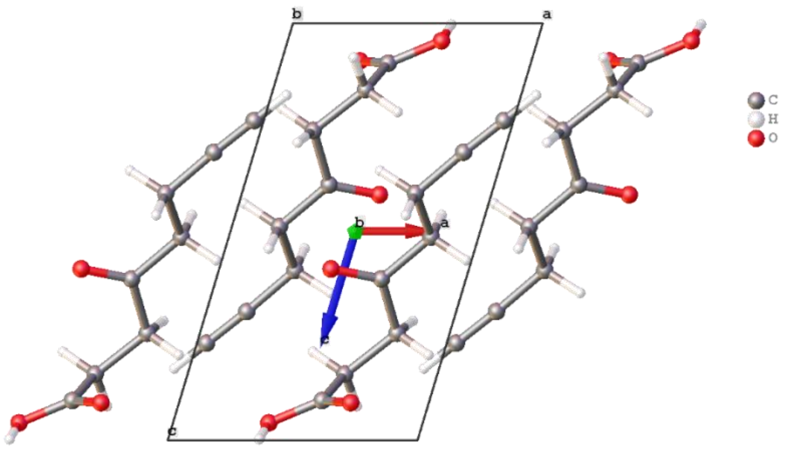

Figure S6: Packing diagram of JJT1123B viewed along the b axis

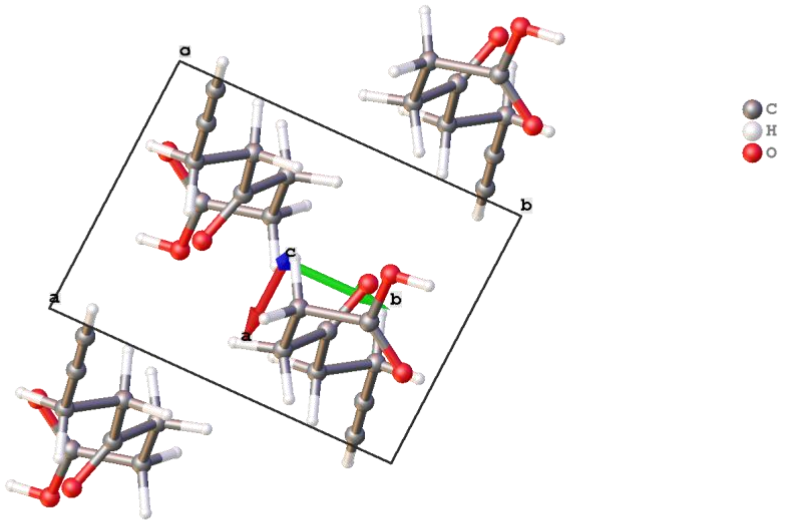

Figure S7: Packing diagram of JJT1123B viewed along the c axis

Figure S8: Data Plots: Diffraction Data

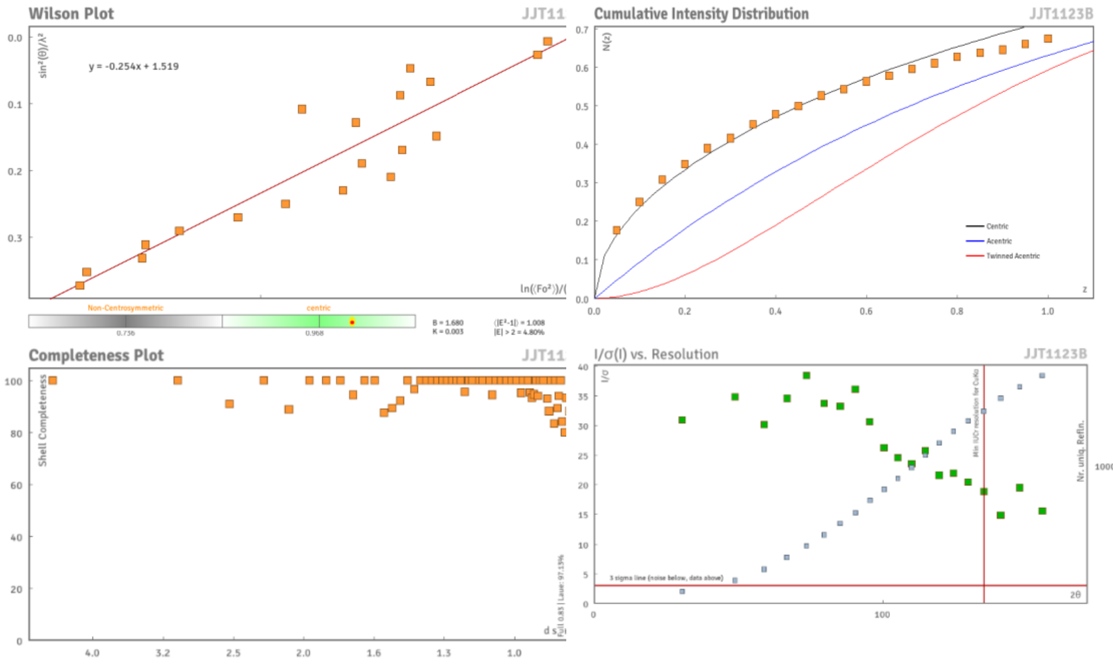

Figure S9: Data Plots: Refinement and Data

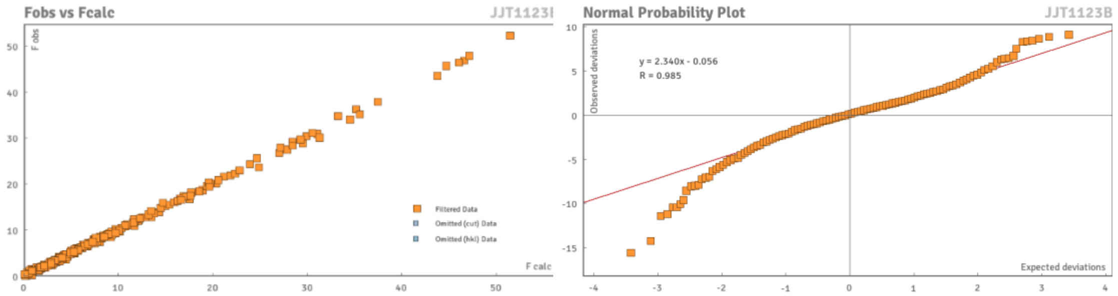

Reflection Statistics

Total reflections (after filtering)	4833	Unique reflections	1605
Completeness	0.925	Mean I/*s*	27.41
hkl_{max} collected	(6, 9, 12)	hkl_{min} collected	(-7, -9, -12)
hkl_{max} used	(6, 9, 12)	hkl_{min} used	(-7, -9, 0)
Lim d_{max} collected	100.0	Lim d_{min} collected	0.77
d_{max} used	9.47	d_{min} used	0.78
Friedel pairs	611	Friedel pairs merged	1
Inconsistent equivalents	3	R_{int}	0.0196
R_{sigma}	0.0275	Intensity transformed	0
Omitted reflections	0	Omitted by user (OMIT hkl)	0
Multiplicity	(976, 565, 313, 171, 100, 52, 26, 8, 4, 1)	Maximum multiplicity	11
Removed systematic absences	0	Filtered off (Shel/OMIT)	0

Figure S10: Selected Crystal Pictures

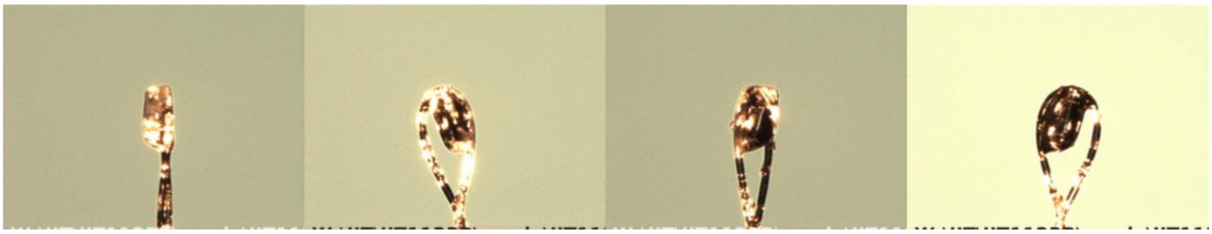

Table S1: Fractional Atomic Coordinates (×10[4]) and Equivalent Isotropic Displacement Parameters (Å^2×10^3) for **JJT1123B**. U_{eq} is defined as 1/3 of the trace of the orthogonalised U_{ij}.

Atom	x	y	z	U_{eq}
O1	5522.7(13)	2765.3(10)	4109.7(8)	22.3(2)
O3	3126.7(14)	851.1(10)	900.9(8)	24.5(2)
O2	6160.6(15)	2361.2(10)	418.7(8)	25.1(2)
C1	4297(2)	2274.6(14)	954.2(10)	21.0(2)
C7	1614.9(19)	1402.5(14)	6911.7(11)	21.1(2)
C6	3051.7(19)	1608.1(14)	5925.1(11)	20.5(2)
C2	3703(2)	4108.7(14)	1588.9(11)	24.3(3)
C4	3387.2(19)	3153.9(13)	3871.2(10)	17.9(2)

Atom	x	y	z	U_{eq}
C8	383(2)	1198.9(16)	7670.5(12)	26.6(3)
C3	2108(2)	3972.0(14)	2569.1(11)	22.3(2)
C5	1897.8(19)	2892.3(13)	4873.3(11)	19.0(2)

Table S2: Anisotropic Displacement Parameters (×10^4) for **JJT1123B**. The anisotropic displacement factor exponent takes the form: -2p^2[h^{2}a*^{2} × U_{11}+ ... +2hka* × b* × U_{12}]$

Atom	U_{11}	U_{22}	U_{33}	U_{23}	U_{13}	U_{12}
O1	18.5(4)	23.7(4)	26.6(4)	4.9(3)	9.0(3)	2.5(3)
O3	28.7(5)	23.7(4)	23.1(4)	0.7(3)	12.2(3)	-6.3(3)
O2	27.5(5)	22.7(4)	27.5(4)	0.4(3)	13.9(3)	-5.5(3)
C1	22.6(6)	24.7(5)	16.0(5)	4.2(4)	5.4(4)	-3.2(4)
C7	20.9(6)	19.4(5)	22.0(5)	3.2(4)	4.4(4)	0.5(4)
C6	19.5(5)	19.4(5)	24.1(5)	3.9(4)	8.4(4)	2.1(4)
C2	28.7(6)	21.9(5)	24.6(5)	6.5(4)	9.7(4)	0.5(4)
C4	18.3(5)	13.0(4)	21.8(5)	-0.5(3)	6.5(4)	-1.0(4)
C8	26.1(6)	30.9(6)	24.6(5)	7.0(4)	8.9(5)	0.2(4)
C3	22.5(6)	21.7(5)	24.3(5)	5.7(4)	7.9(4)	3.4(4)
C5	17.7(5)	17.9(5)	22.2(5)	2.6(4)	7.3(4)	1.4(4)

Table S3: Bond Lengths in Å for **JJT1123B**.

Atom	Atom	Length/Å
O1	C4	1.2160(13)
O3	C1	1.2214(13)
O2	C1	1.3182(12)
C1	C2	1.5017(15)
C7	C6	1.4661(14)
C7	C8	1.1886(15)
C6	C5	1.5286(14)
C2	C3	1.5212(14)
C4	C3	1.5092(14)
C4	C5	1.5096(13)

Table S4: Bond Angles in ° for **JJT1123B**.

Atom	Atom	Atom	Angle/°
O3	C1	O2	123.27(9)
O3	C1	C2	123.45(9)
O2	C1	C2	113.24(9)
C8	C7	C6	177.52(11)
C7	C6	C5	112.00(8)
C1	C2	C3	113.01(8)
O1	C4	C3	121.44(9)
O1	C4	C5	122.05(9)
C3	C4	C5	116.50(8)
C4	C3	C2	112.95(9)
C4	C5	C6	112.67(8)

Table S5: Torsion Angles in ° for **JJT1123B**.

Atom	Atom	Atom	Atom	Angle/°
O1	C4	C3	C2	4.06(14)
O1	C4	C5	C6	-17.17(13)
O3	C1	C2	C3	18.97(15)
O2	C1	C2	C3	-163.08(9)
C1	C2	C3	C4	65.31(12)
C7	C6	C5	C4	178.65(8)
C3	C4	C5	C6	164.36(8)
C5	C4	C3	C2	-177.47(8)

Table S6: Hydrogen Fractional Atomic Coordinates (×10^4) and Equivalent Isotropic Displacement Parameters (Å^2×10^3) for **JJT1123B**. U_{eq} is defined as 1/3 of the trace of the orthogonalised U_{ij}.

Atom	x	y	z	U_{eq}
H2	6352.69	1315.87	27.98	38
H6A	3173.1	392.26	5400.92	25
H6B	4737.57	2094.69	6465.16	25
H2A	2841.31	4784.56	818.81	29
H2B	5256.08	4819.28	2124.46	29
H8	-601.2	1036.25	8277.05	32
H3A	1621.78	5210.1	2861.32	27
H3B	589.73	3211.57	2046.19	27
H5A	225.85	2389.01	4320.38	23
H5B	1737.73	4097.12	5400.6	23

Table S7: Hydrogen Bond information for **JJT1123B**.

D	H	A	d(D-H)/Å	d(H-A)/Å	d(D-A)/Å	D-H-A/deg
O2	H2	O3[1]	0.84	1.81	2.6489(10)	175.6

———

[1]1-x,-y,-z

Citations

CrysAlisPro (ROD), Rigaku Oxford Diffraction, Poland (?).

CrysAlisPro Software System, Rigaku Oxford Diffraction, (2023).

O.V. Dolomanov and L.J. Bourhis and R.J. Gildea and J.A.K. Howard and H. Puschmann, Olex2: A complete structure solution, refinement and analysis program, *J. Appl. Cryst.*, (2009), **42**, 339-341.

Sheldrick, G.M., Crystal structure refinement with ShelXL, *Acta Cryst.*, (2015), **C71**, 3-8.

Sheldrick, G.M., ShelXT-Integrated space-group and crystal-structure determination, *Acta Cryst.*, (2015), **A71**, 3-8.

Figure S11: ^{1}H and ^{13}C{^{1}H} NMR Spectra of compound 4

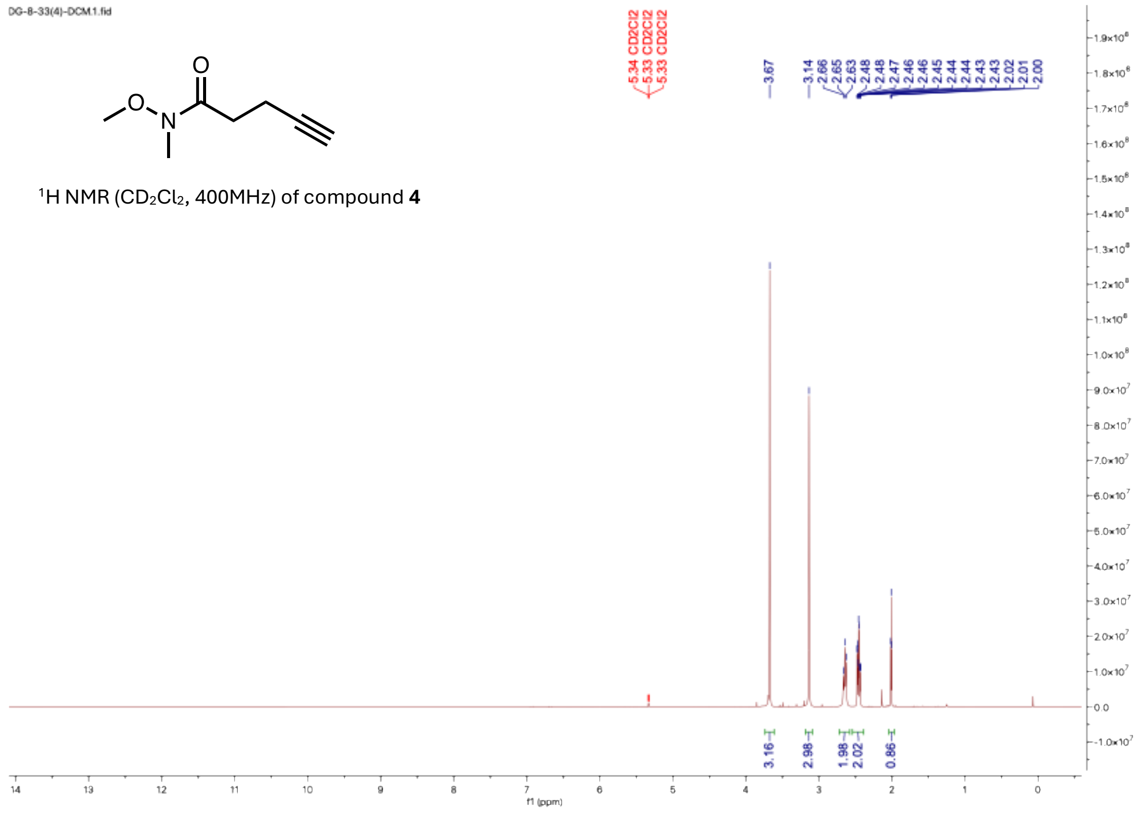

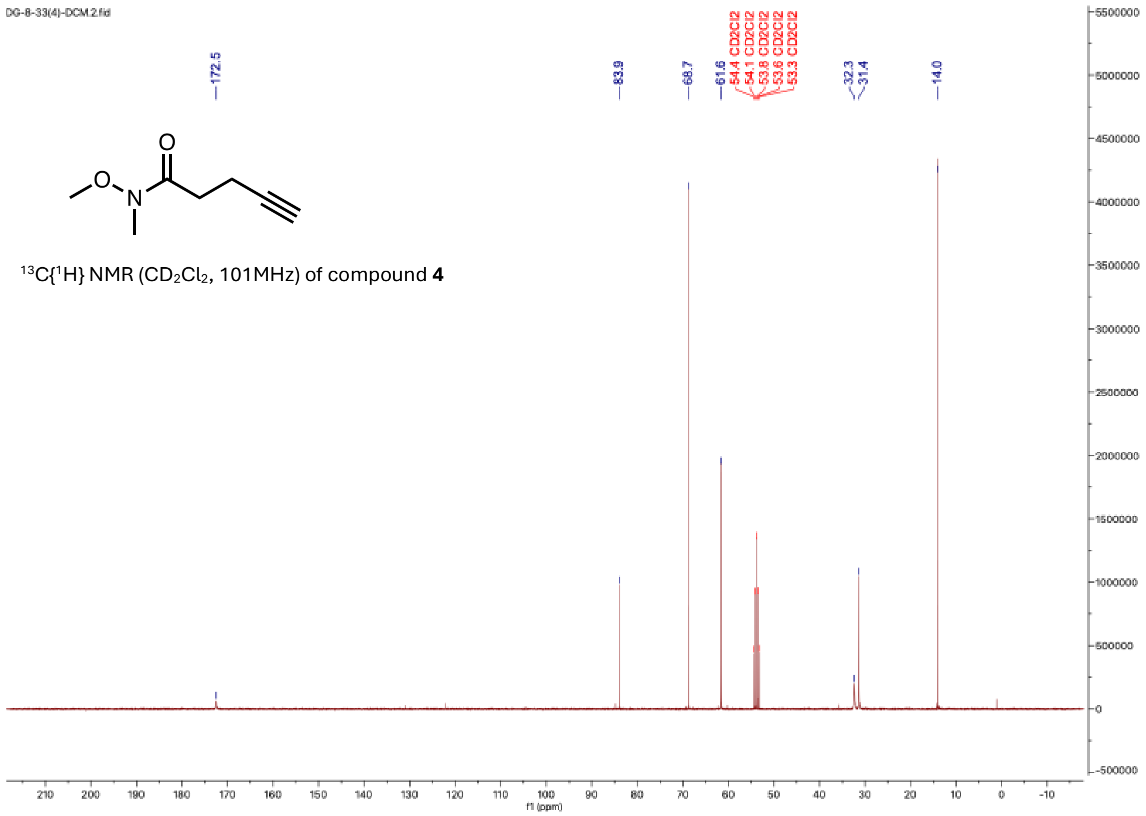

Figure S12: ^{1}H and ^{13}C{1H} NMR Spectra of compound 6

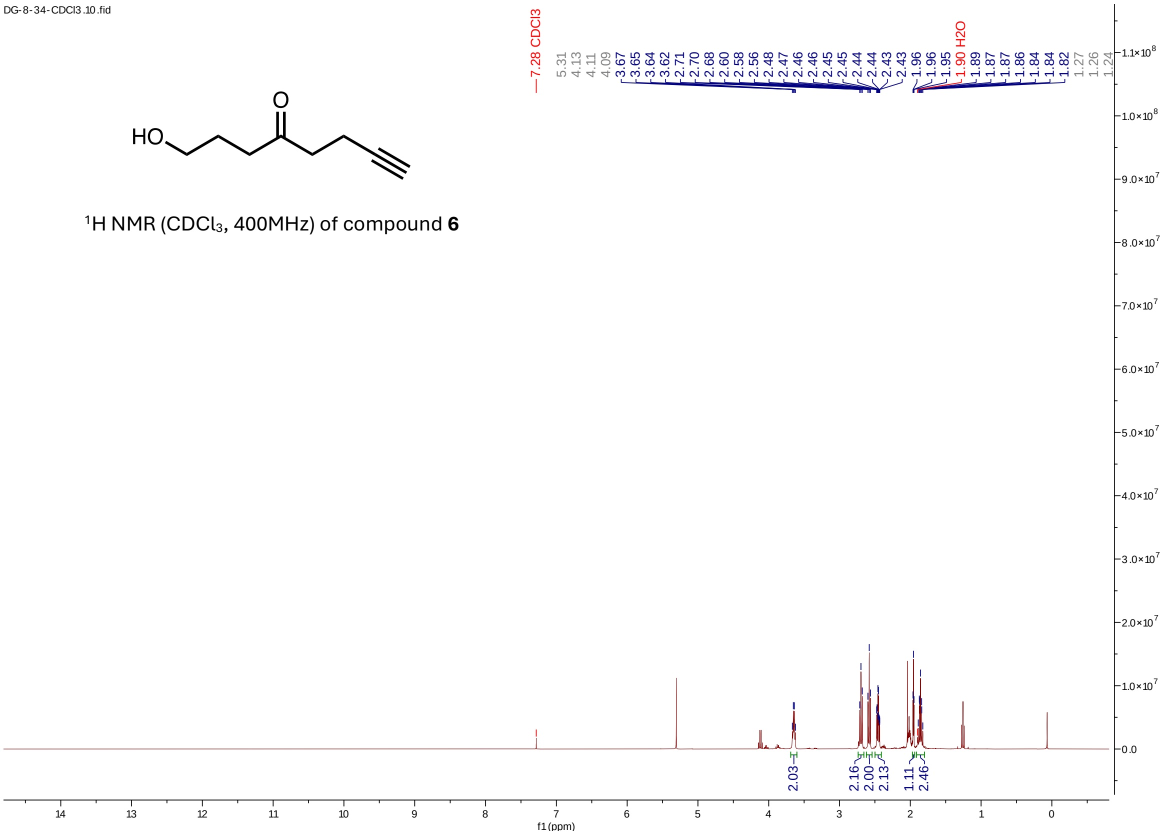

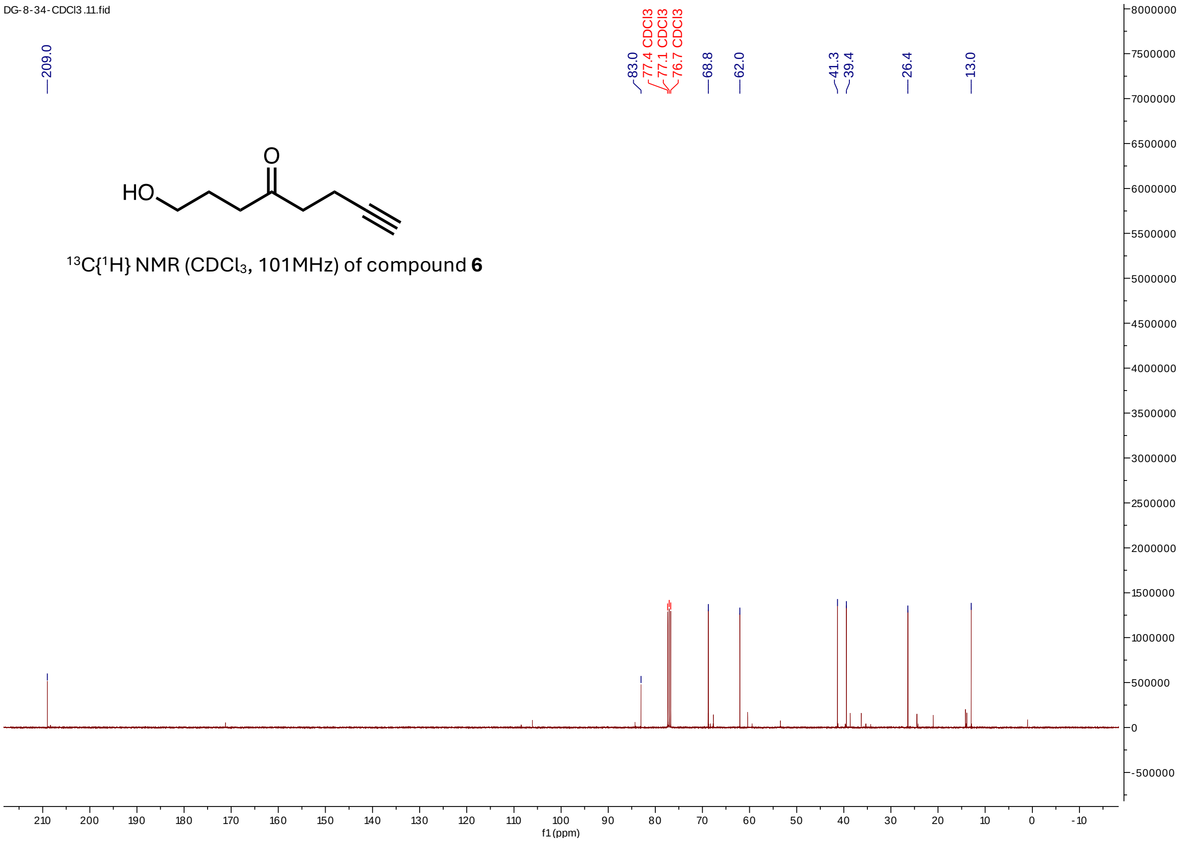

Figure S13: ^{1}H and $^{13}C\{^{1}H\}$ NMR Spectra of compound 6 in acetone-d_6

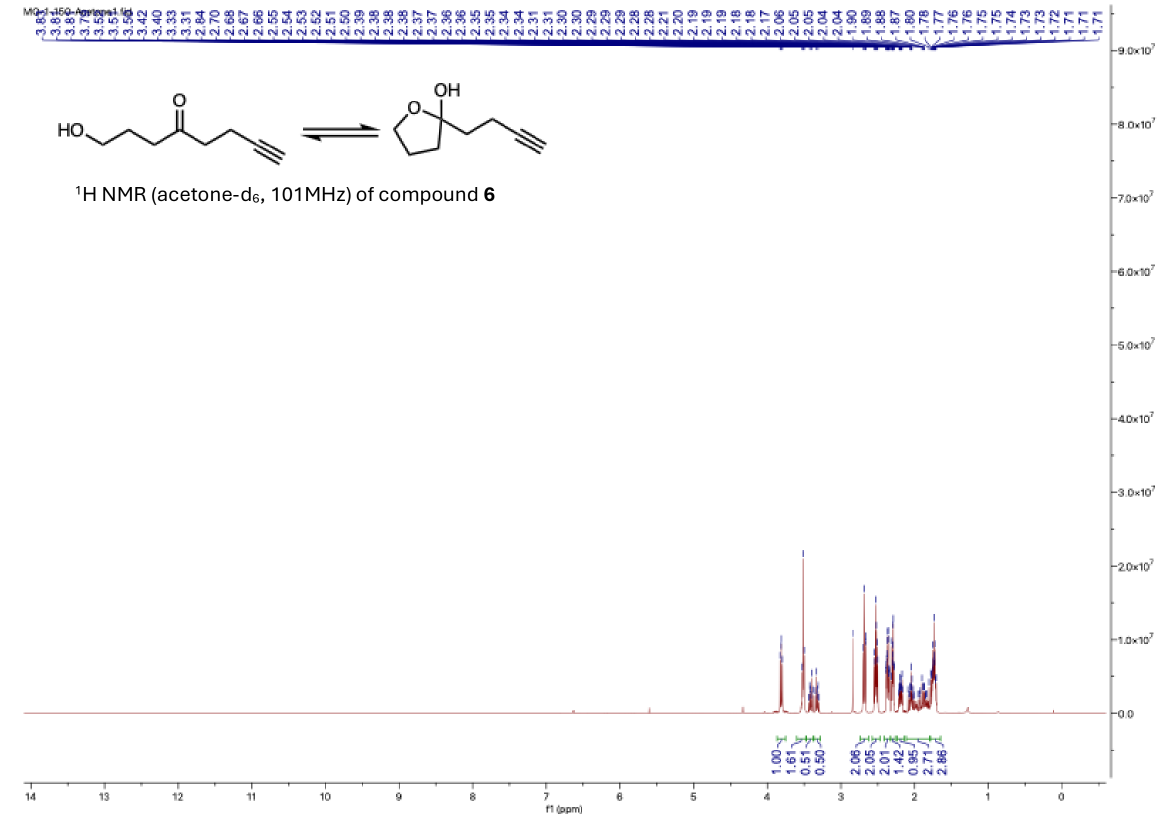

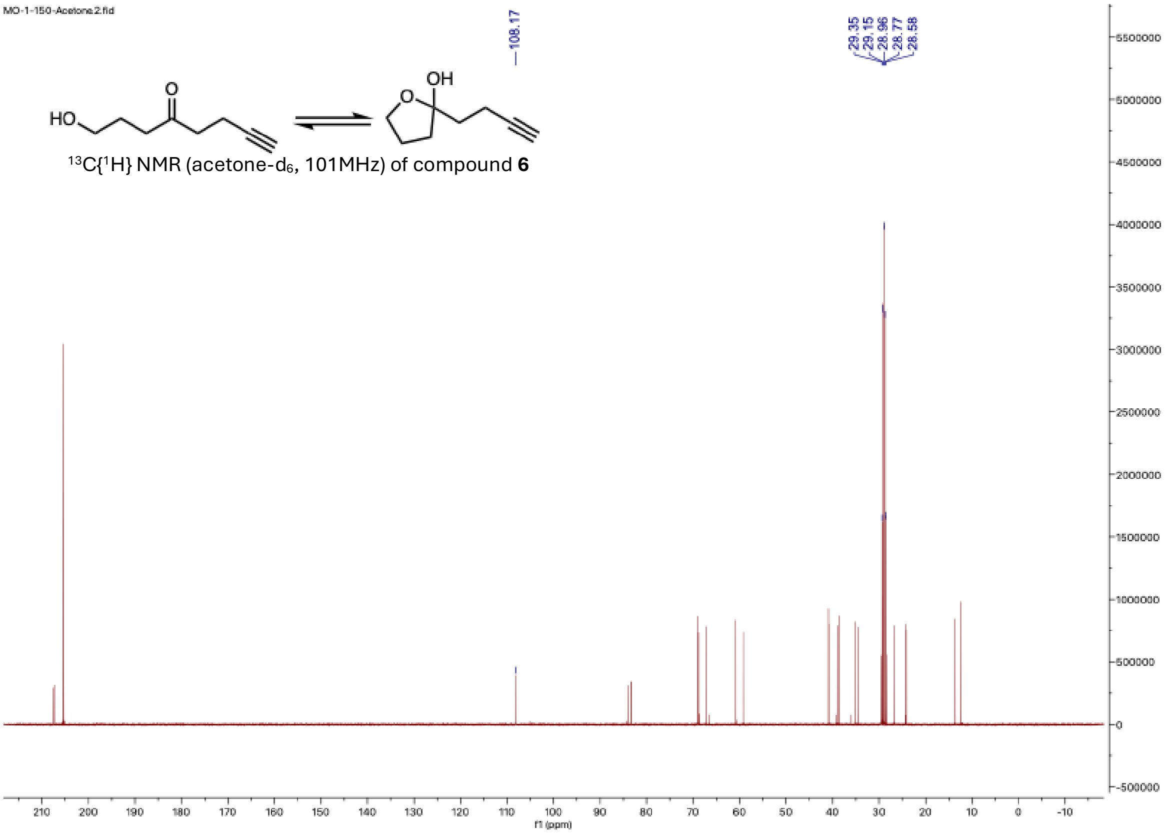

Figure S14: ^{1}H and ^{13}C{^{1}H} NMR Spectra of compound 7

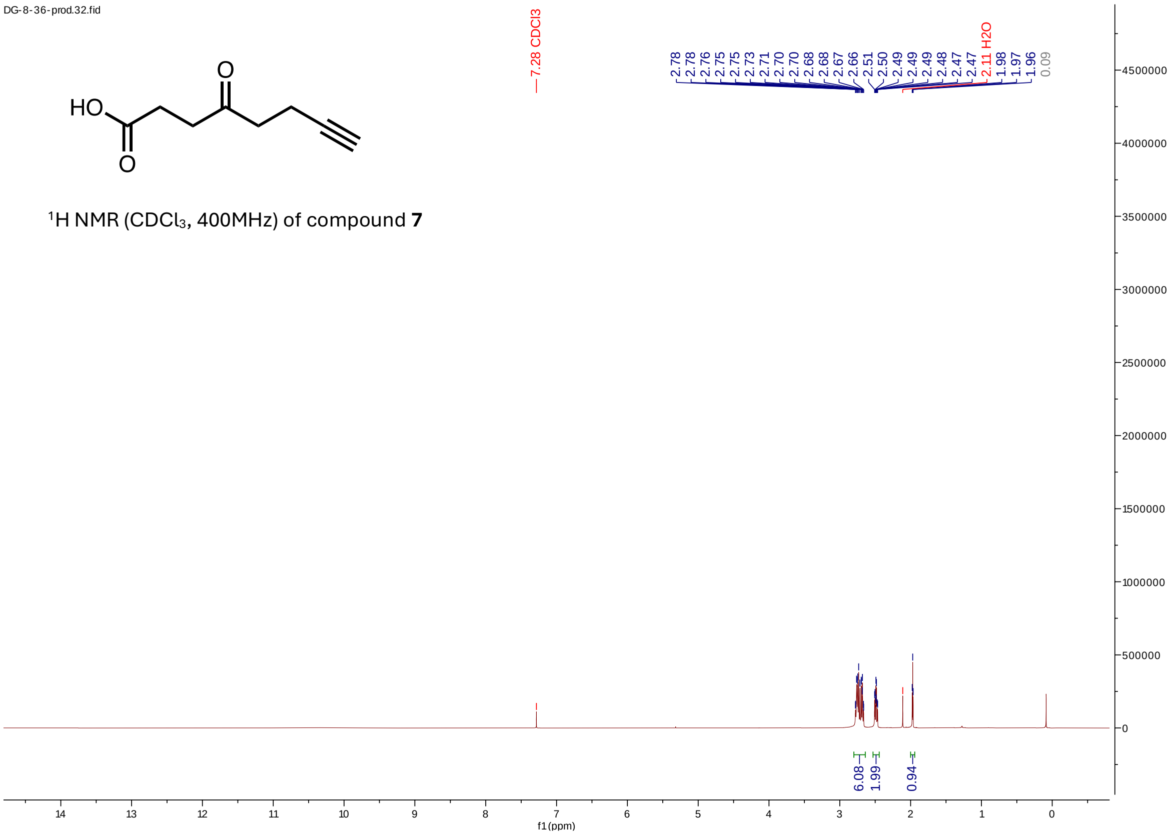

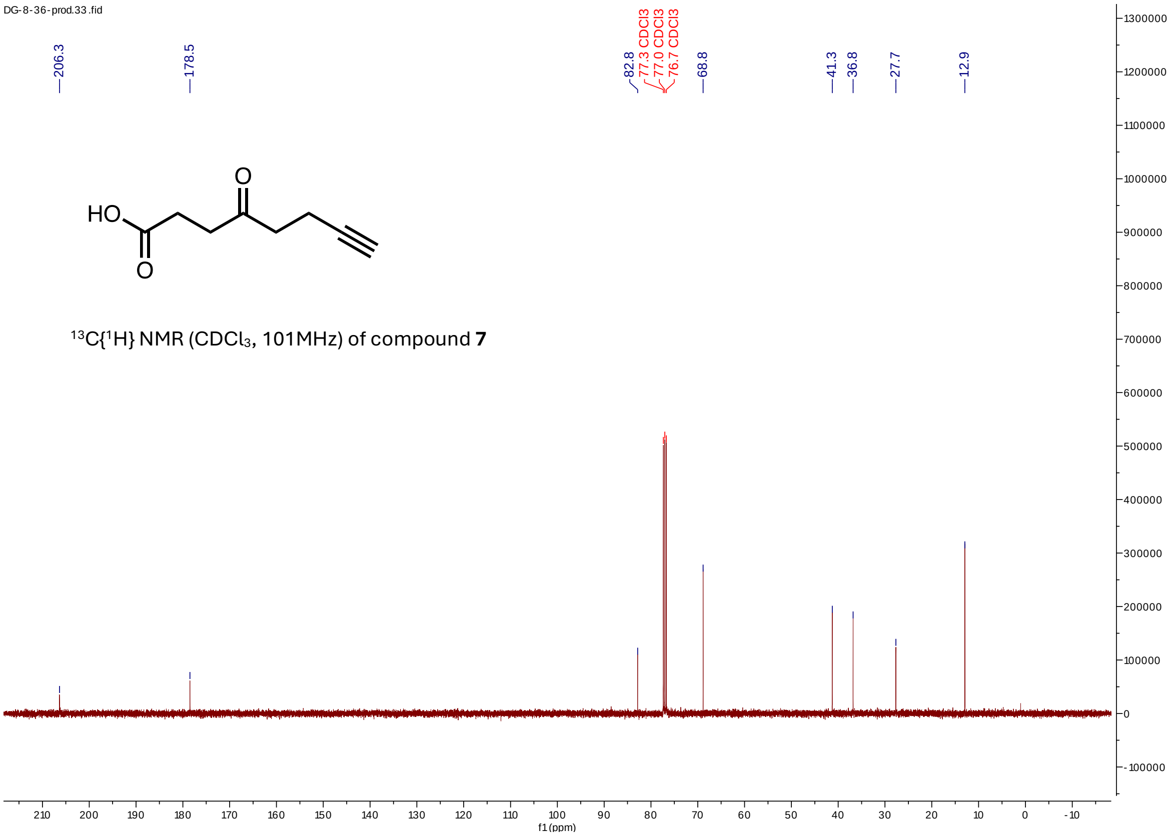

Figure S15: ^{1}H and ^{13}C{^{1}H} NMR Spectra of compound 7b

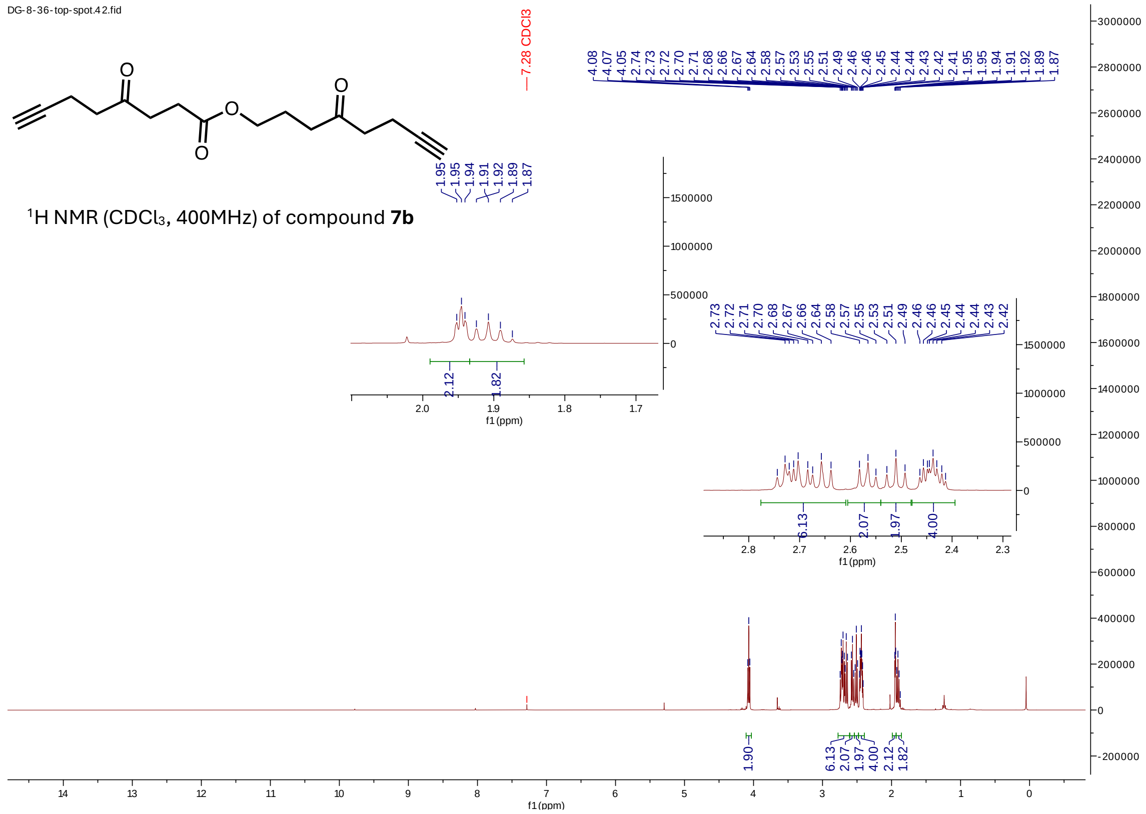

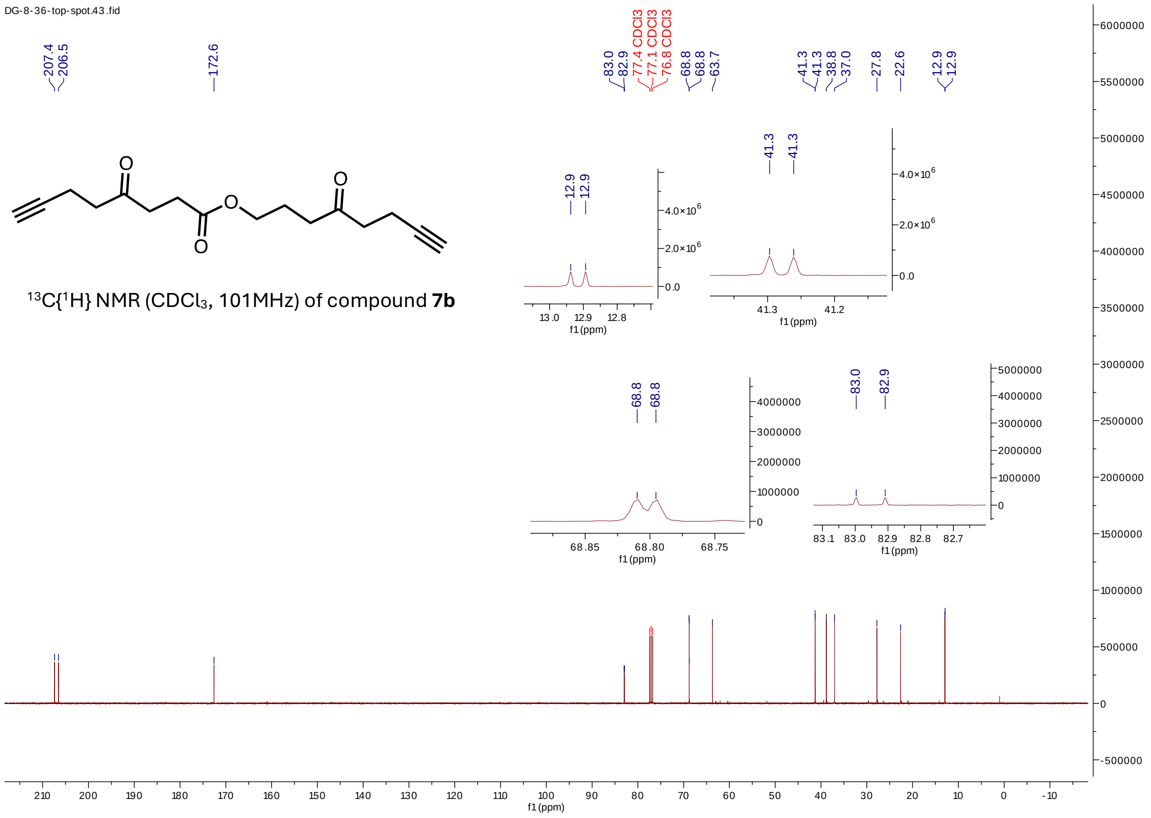

Figure S16: ^1H and $^{13}C\{^1H\}$ NMR Spectra of compound 1

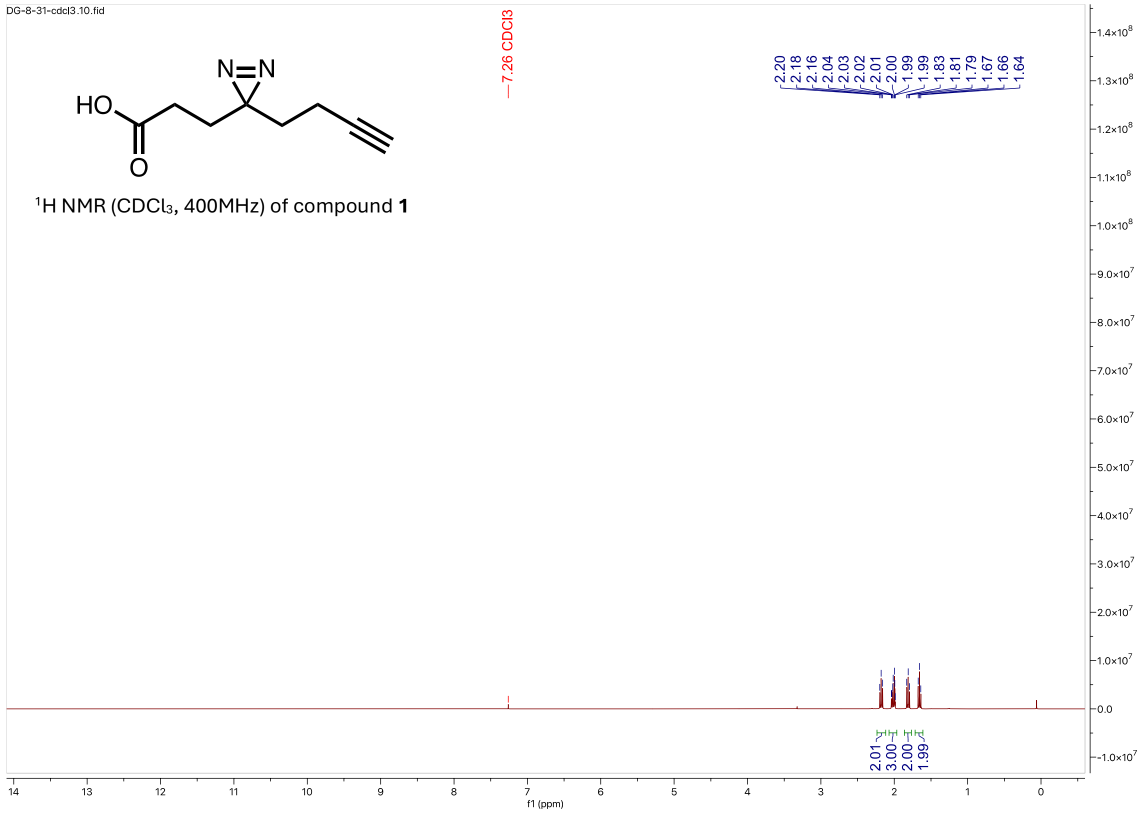

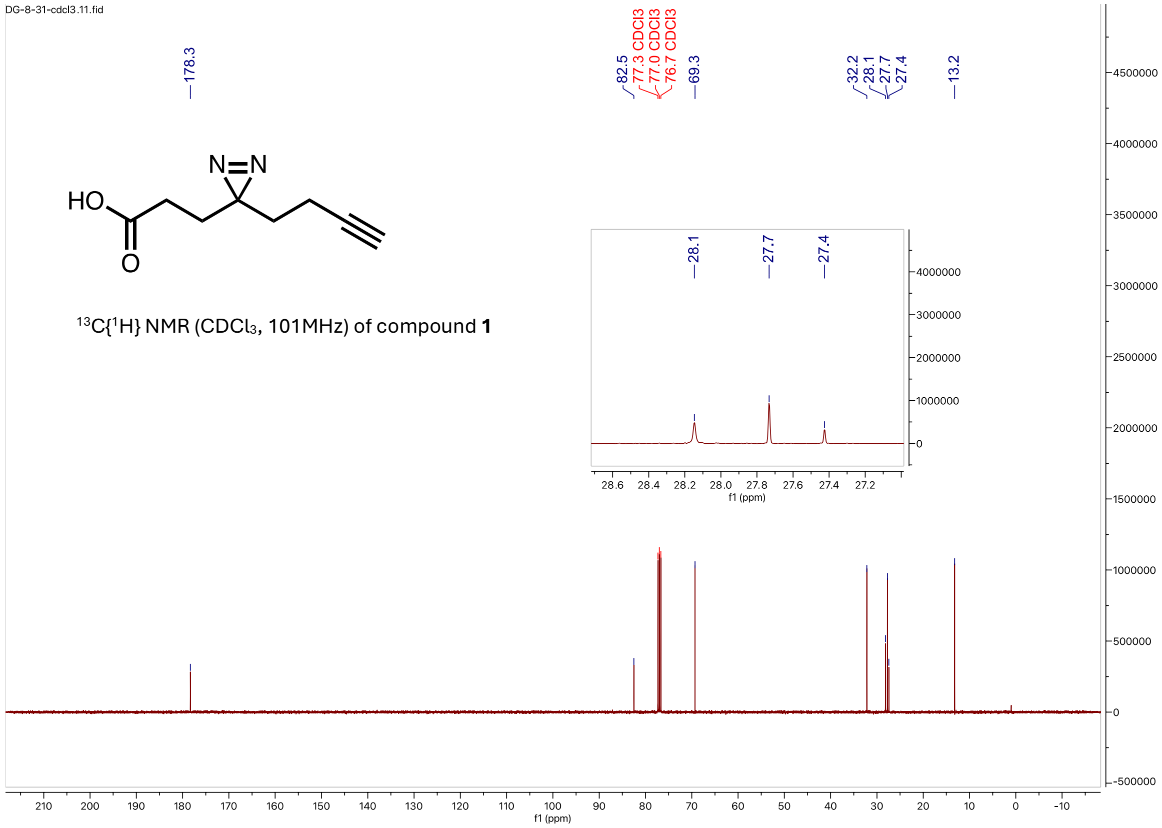

Figure S17: ^{1}H and ^{13}C{^{1}H} NMR Spectra of compound 8

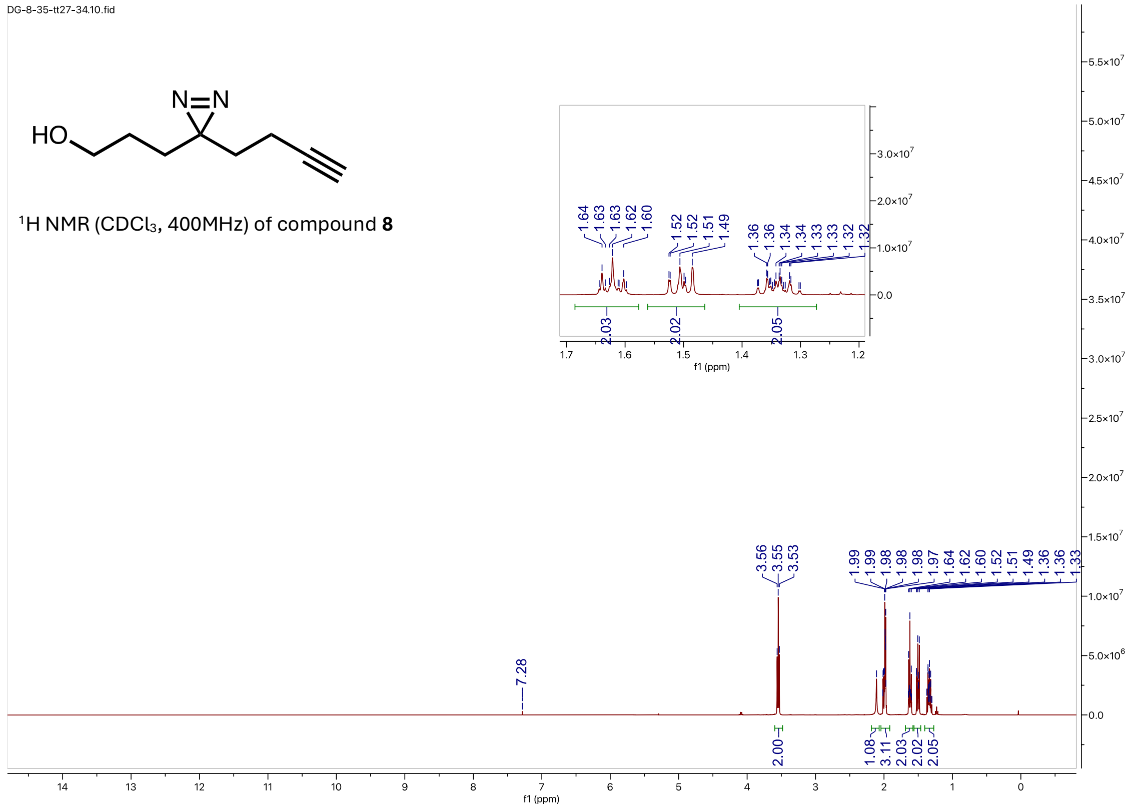

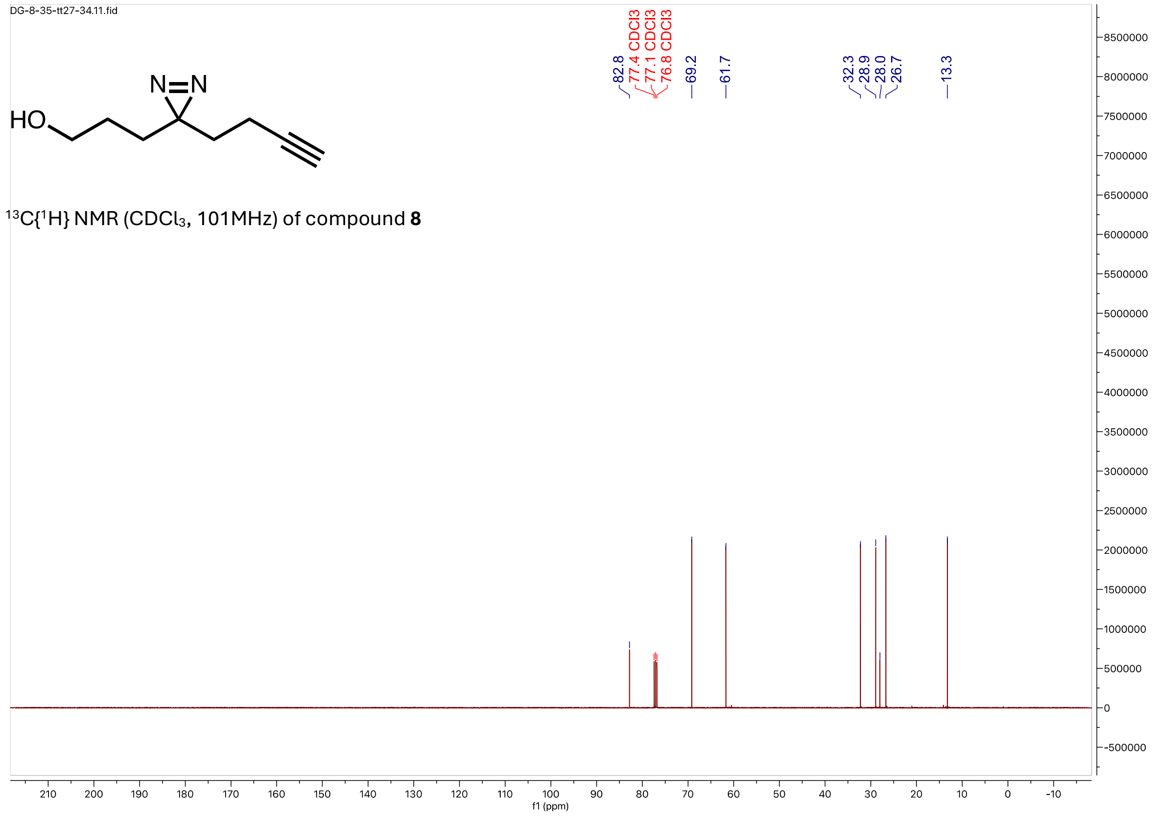

Figure S18: ^{1}H and $^{13}C\{^{1}H\}$ NMR Spectra of compound 9

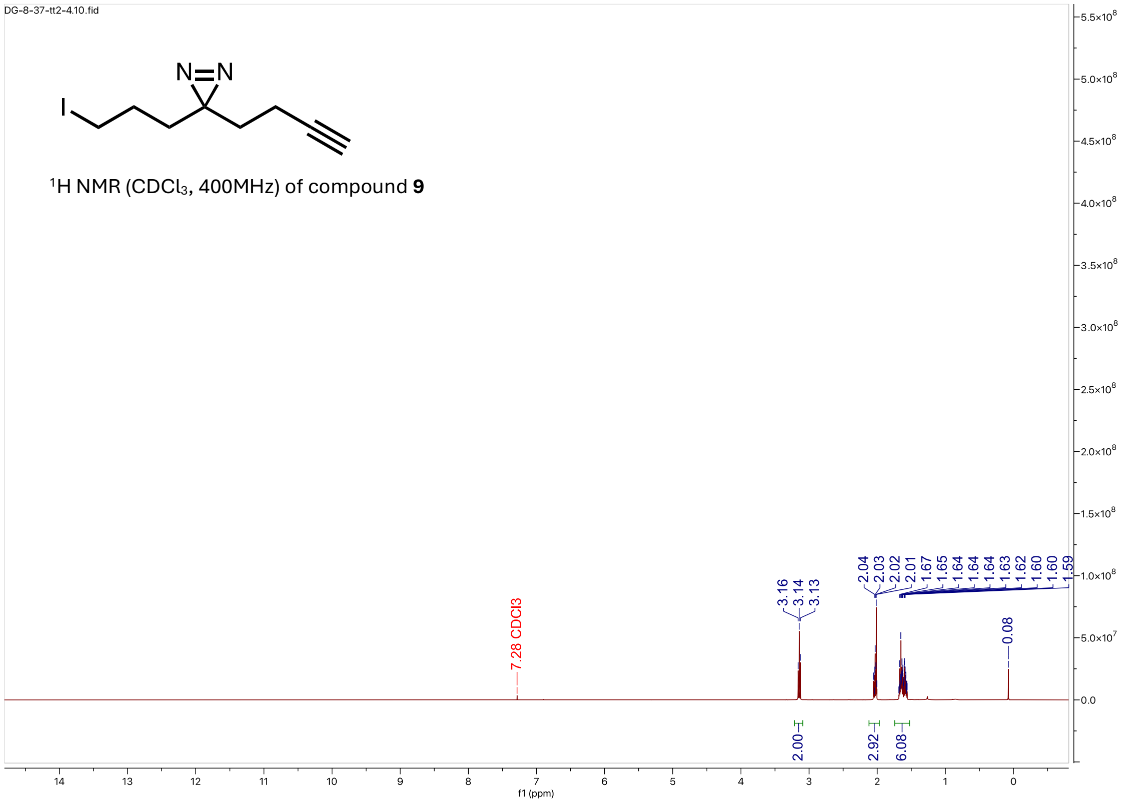

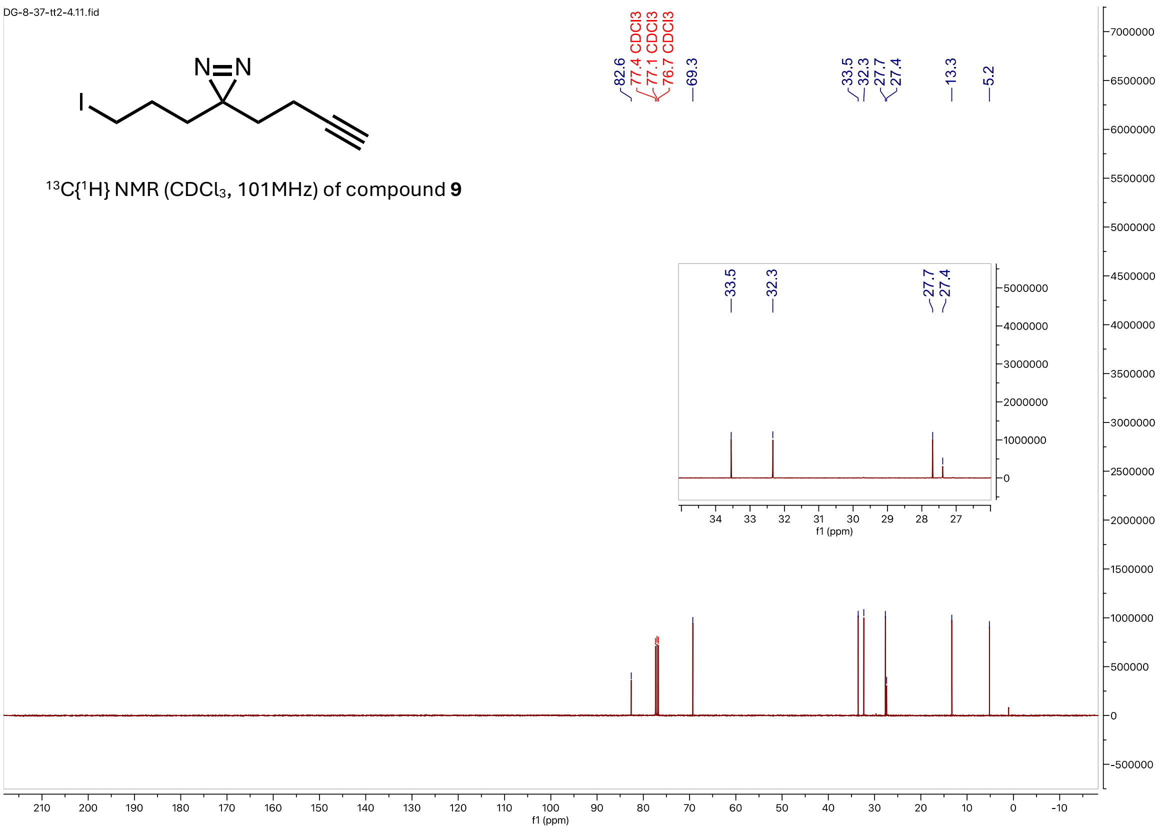

Figure S19: ^{1}H and ^{13}C{^{1}H} NMR Spectra of compound 11

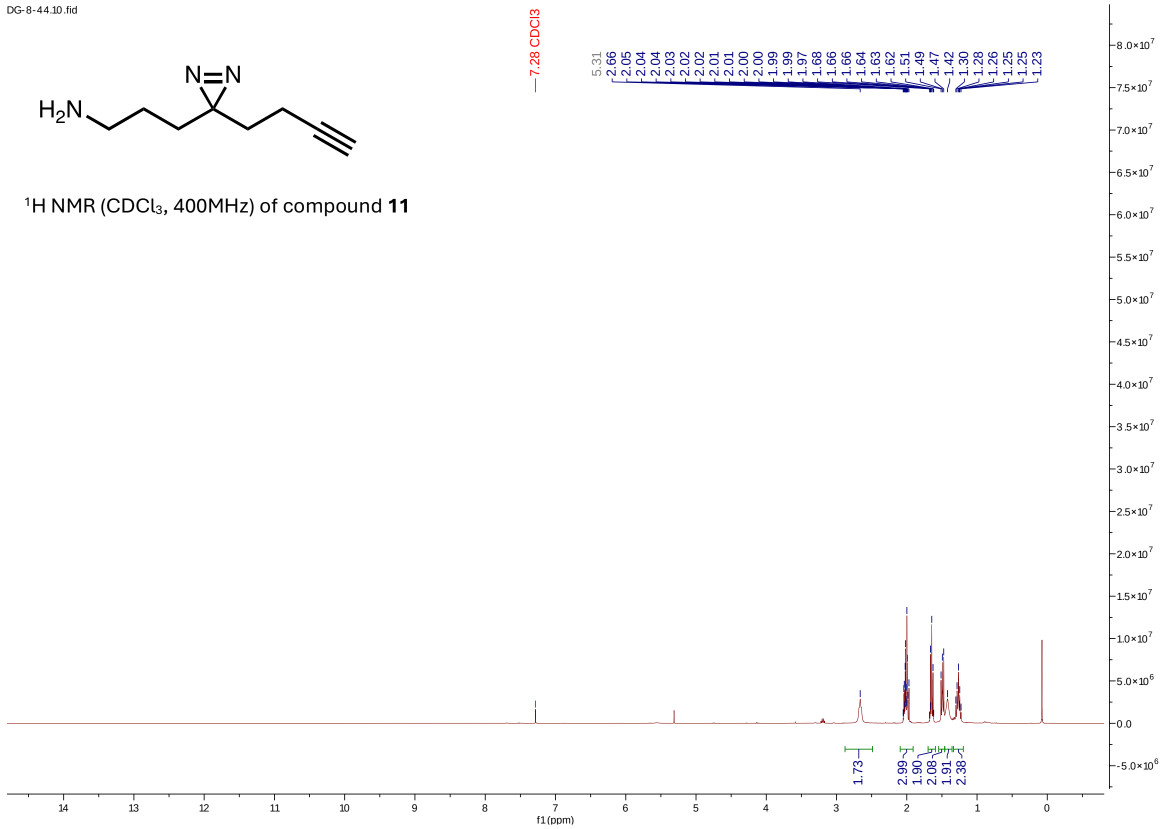

^{1}H NMR ($CDCl_3$, 400MHz) of compound **11**

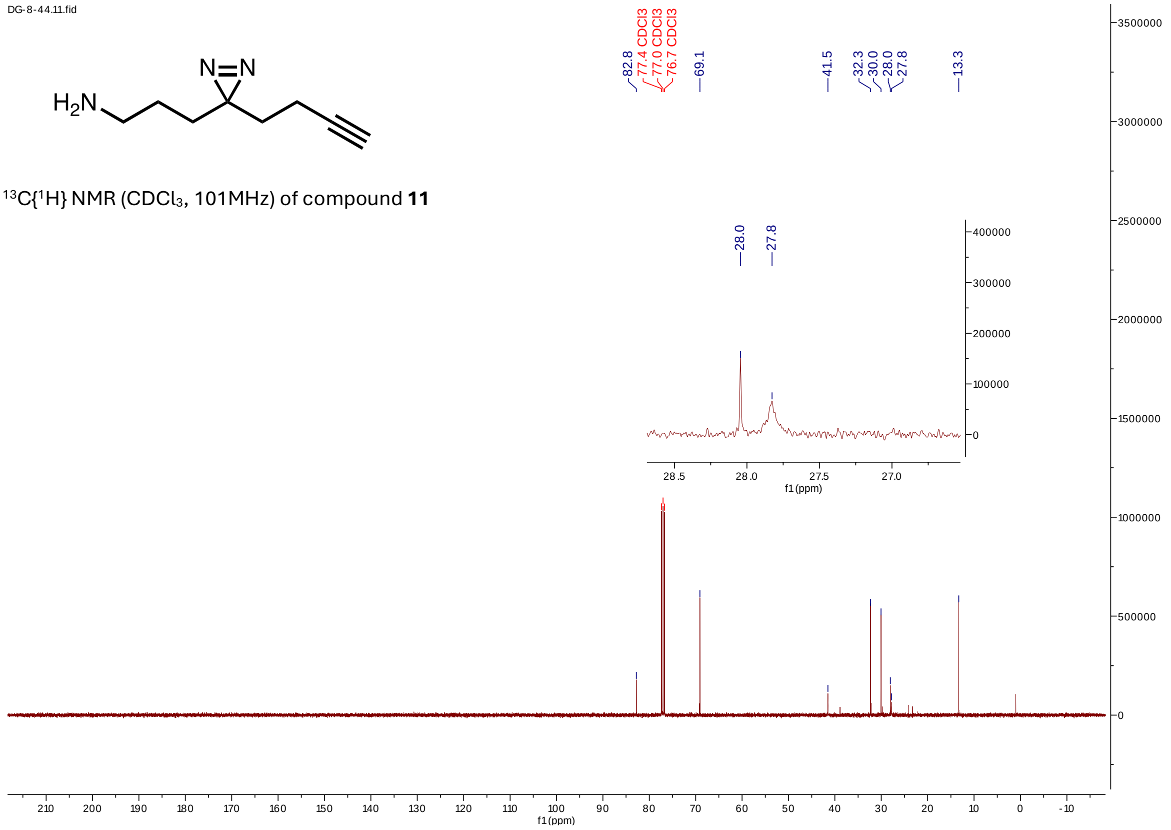

^{13}C{^{1}H} NMR ($CDCl_3$, 101MHz) of compound **11**
